# Supplementary material for: CPSF3 Promotes Pre-mRNA Splicing and Prevents CircRNA Cyclization in Hepatocellular Carcinoma
Source: Cancers (Basel). 2023 Aug 11;15(16):4057. doi: 10.3390/cancers15164057 (PMC10452738; doi:10.3390/cancers15164057)
Supplement: Supplementary file 1 [file cancers-15-04057-s001.zip › supplementary file S2.pdf]

Ethics Committee of Xiangya College of Pharmacy, Central South University

Approval documents for scientific research projects

NO. 202251

Project Name	CPSF3 modulates the balance of circular and linear transcripts in hepatocellular carcinoma				
Specialty	Pharmacology	Responsibility	Direct	Principal Investigator	Qubo Zhu
Classification	1☑ 2☐ 3☑	Duration of study		2020.1-2023.12	
Study Type	Basic Research				
Research Units	Central South University				
Comment	☑ **Approval** ☐Approval after modification ☐Retrial after modification ☐disapproval ☐Suspension or termination of the study				

Main Component:

In this study, a subcutaneous tumor model of nude mice was constructed by subcutaneous injection of hepatocellular carcinoma cells under the armpit, and the effect of CPSF3 inhibitor JTE-607 on the tumorigenic ability of cancer cells in vivo was studied. Subsequently, the mice were killed by cervical dislocation, and subcutaneous tumors were obtained for detection and analysis. During the experiment, the team will carry out research work in strict accordance with the ethical regulations of experimental animals, follow the 3R principle, and ensure that animals are treated well during breeding, application, and transportation, and use Baoding and euthanasia when necessary to treat experiments with humanity animal.

Five cancer samples were obtained from the Third Xiangya Hospital of Central South University and stored in liquid nitrogen until analyzed. Extract RNA and protein for related experiments to study the expression level of CPSF3 in liver cancer samples. All experiments were conducted in accordance with the provisions of the Declaration of Helsinki and good clinical practice guidelines.

Ethical review Opinion:

After deliberation by the ethics committee of our hospital, the experimental design and program of the study fully considered the principles of safety and fairness, protected the rights of animals, and would minimize the pain and tension of animals. The sample acquisition process complies with relevant regulations, and there is no conflict of interest in the research content and research results.

Note: Study Type: 1= Animal Experiment; 2= Experiments on animal tissues or cells; 3=Others

Ethics Committee of Xiangya College of Pharmacy,Central South University

2022.12.21

中南大学湘雅药学院伦理委员会
