# Supplementary material for: CPSF3 Promotes Pre-mRNA Splicing and Prevents CircRNA Cyclization in Hepatocellular Carcinoma
Source: Cancers (Basel). 2023 Aug 11;15(16):4057. doi: 10.3390/cancers15164057 (PMC10452738; doi:10.3390/cancers15164057)

Fig2. E

CPSF3

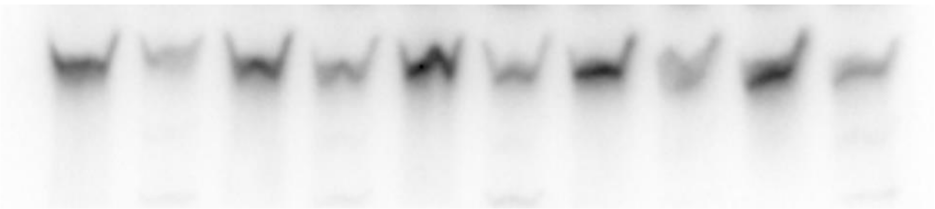

Actin

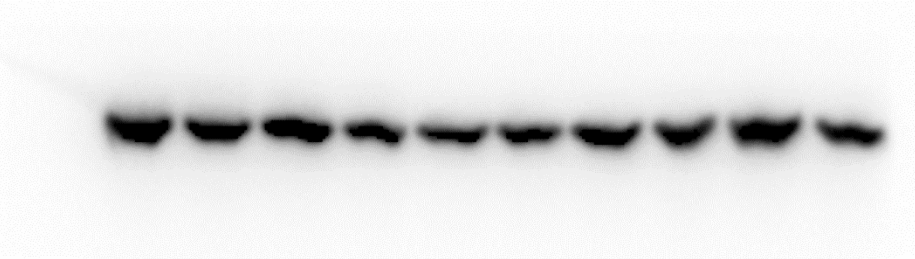

Fig2. F

CPSF3

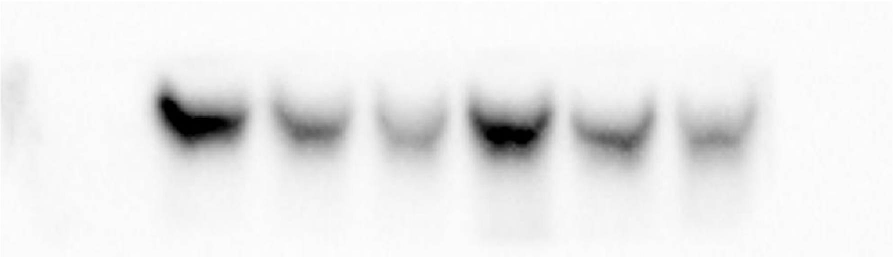

Actin

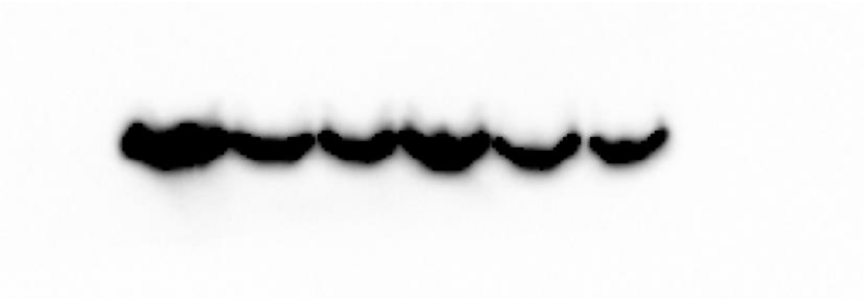

Fig3. A

CPSF3

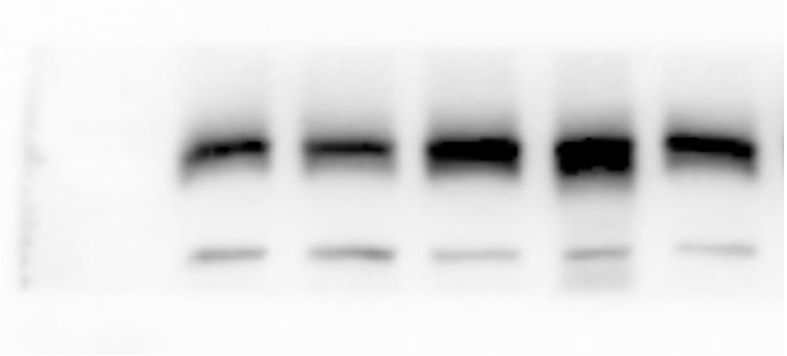

Actin

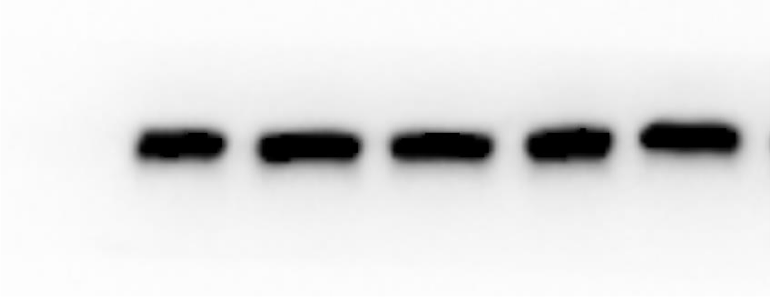

Fig3. B

CPSF3

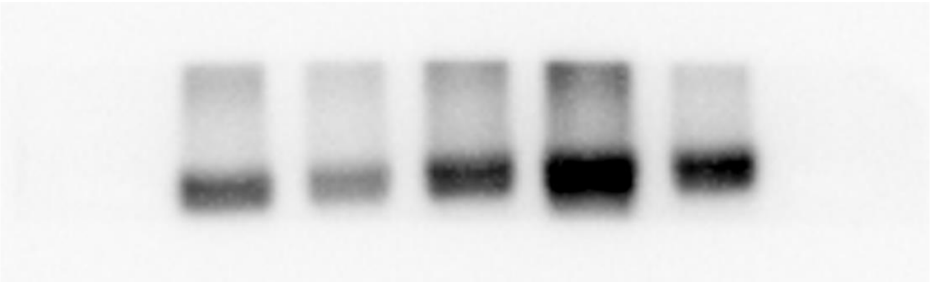

Actin

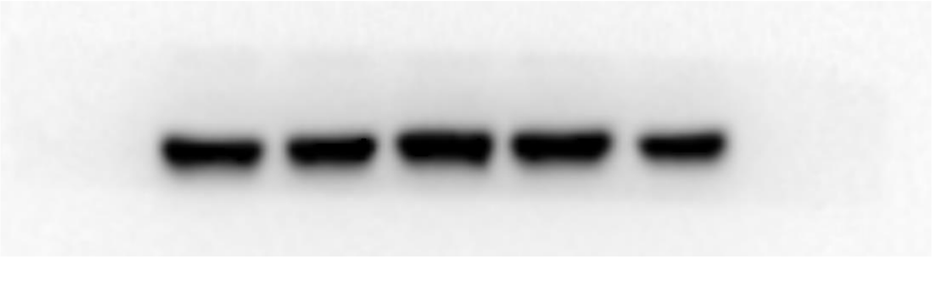

Fig6. B

hsa_circ_0001380

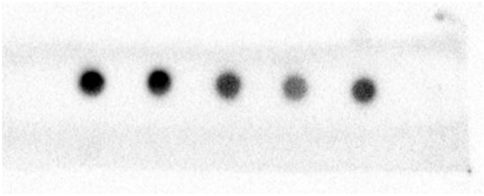

hsa_circ_0078607

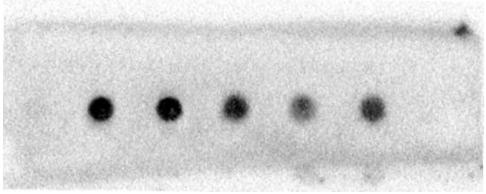

hsa_circ_0008305

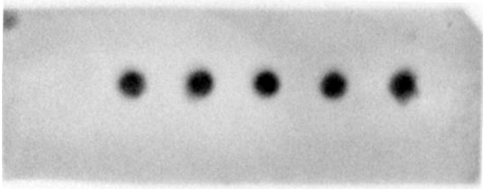

β-actin

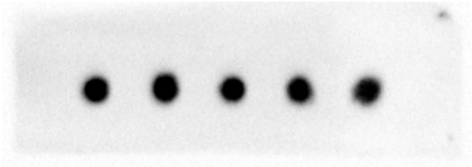

Fig6. D

UBXN7

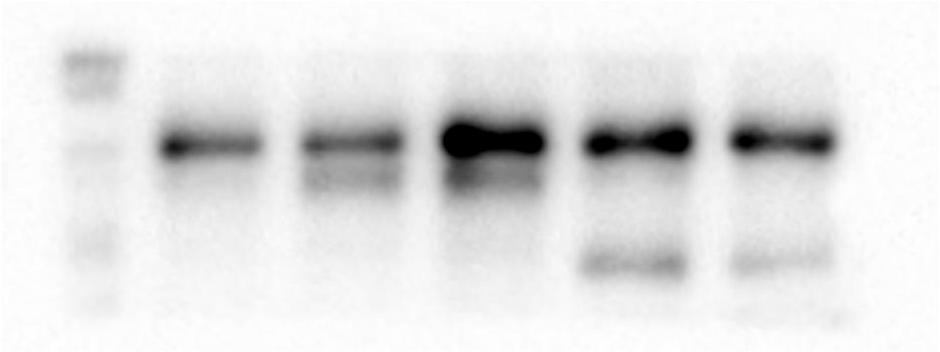

SLC22A3

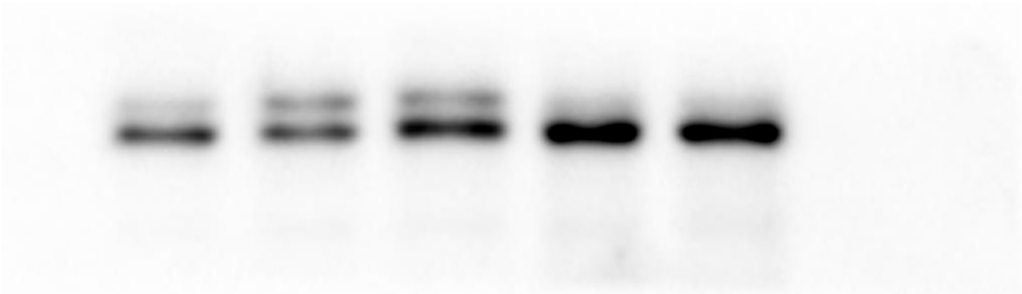

Actin

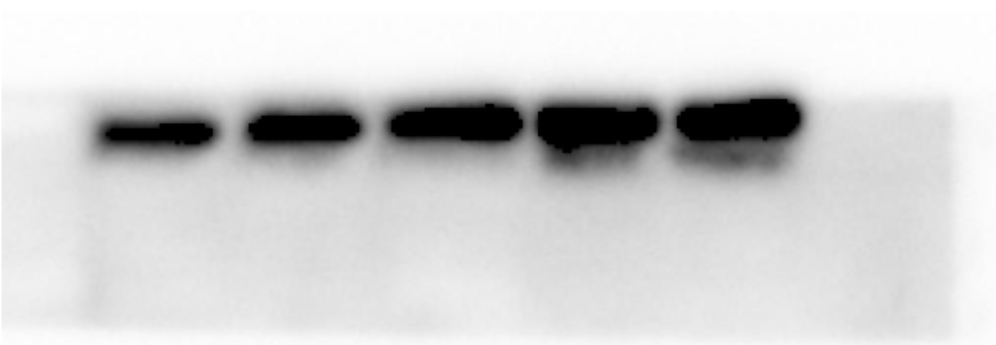

Fig6. H

E2F1

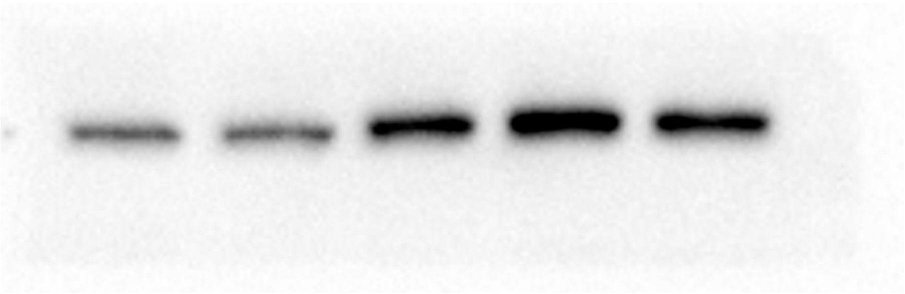

NCAPG2

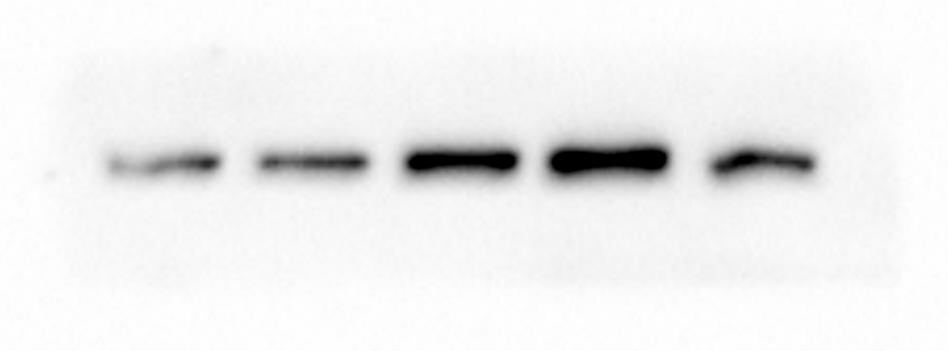

CPSF3

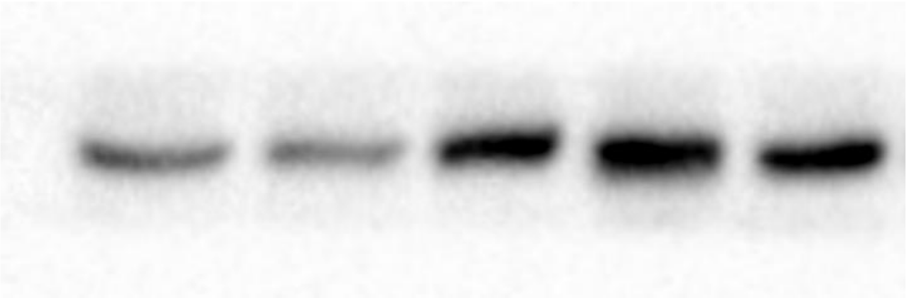

Actin

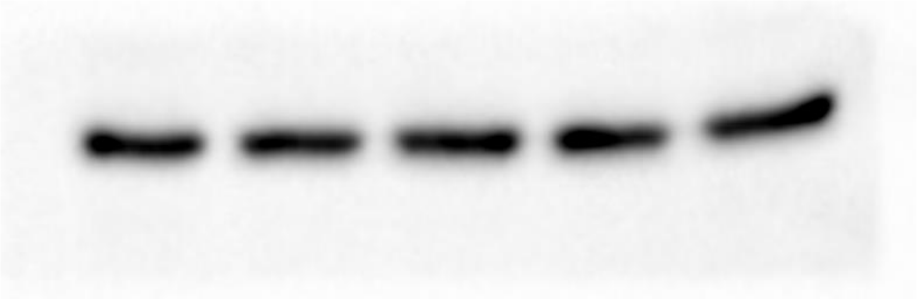

Fig7. E-left

E2F1

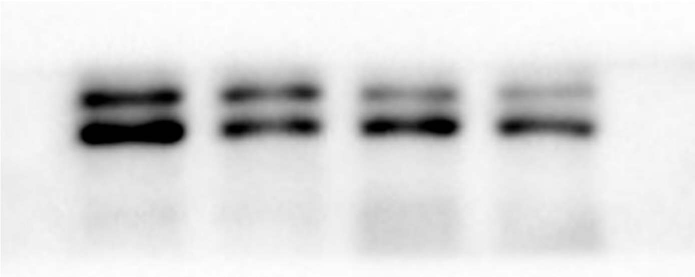

NCAPG2

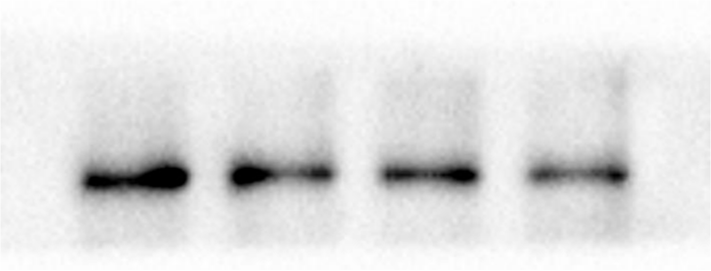

GAPDH

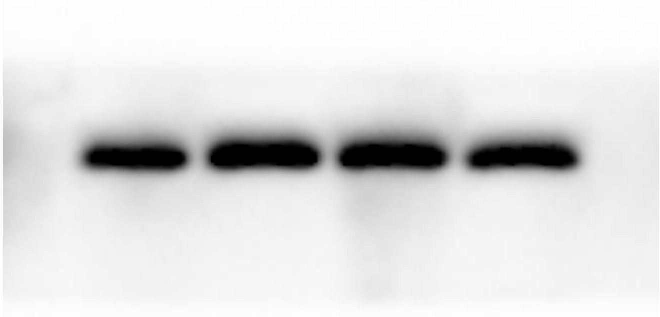

Fig7. E-right

E2F1

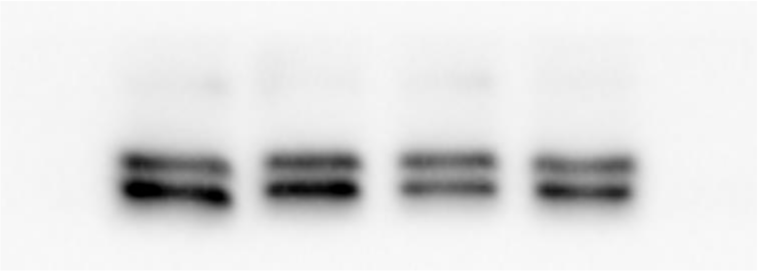

NCAPG2

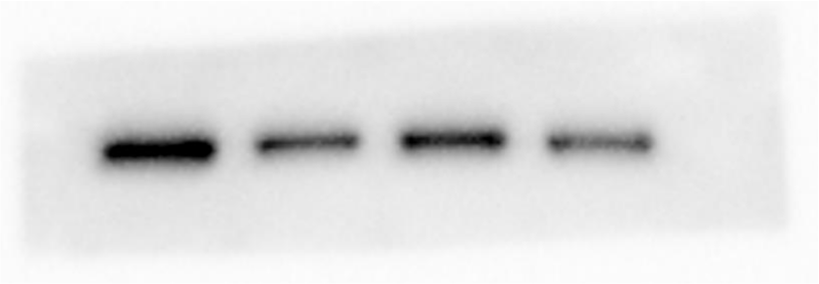

GAPDH

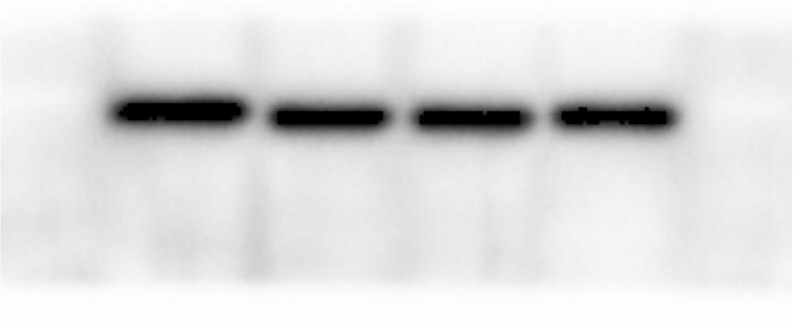

Fig7. F-left

UBXN7

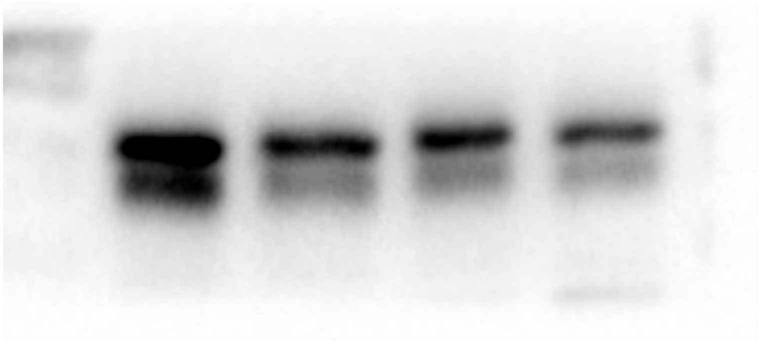

SCL22A3

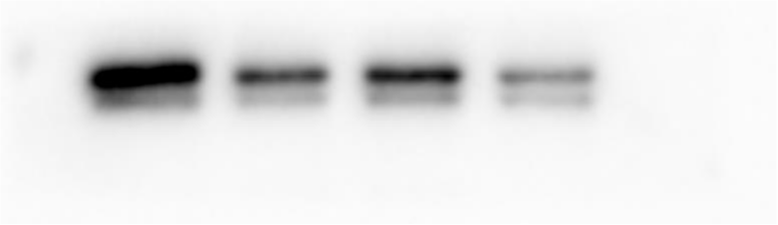

GAPDH

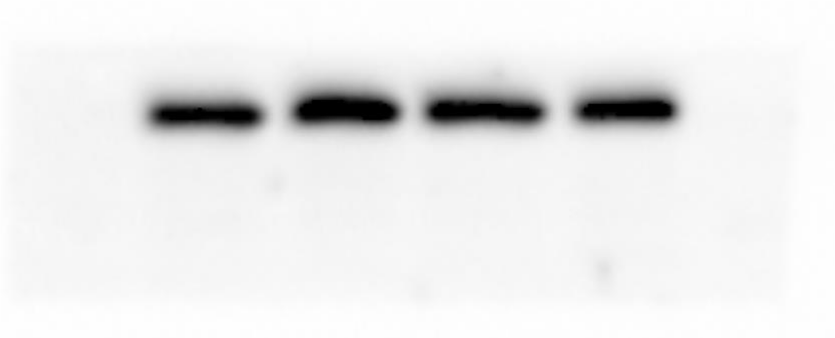

Fig7. F-right

UBXN7

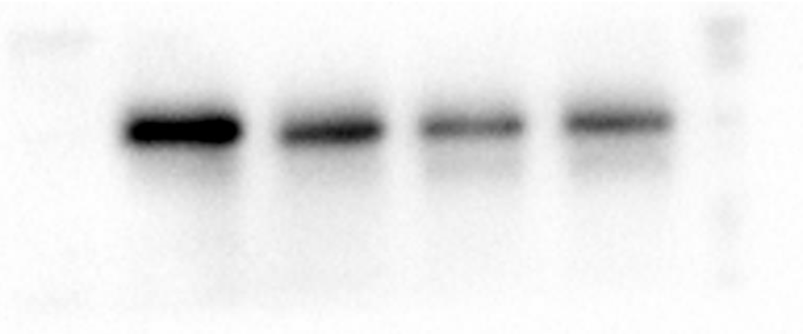

SLC22A3

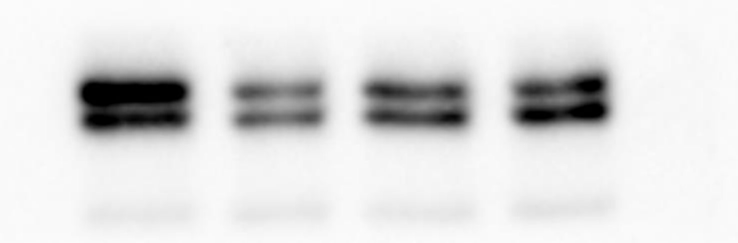

GAPDH

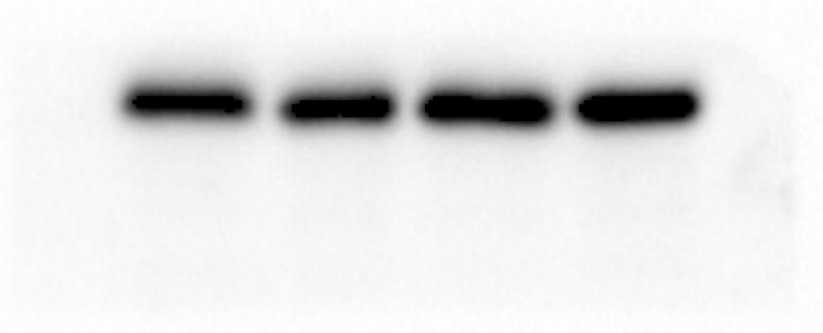

Supplement: Supplementary file 1 [file cancers-15-04057-s001.zip › supplementary file S9.pdf]
