# Supplementary material for: CPSF3 Promotes Pre-mRNA Splicing and Prevents CircRNA Cyclization in Hepatocellular Carcinoma
Source: Cancers (Basel). 2023 Aug 11;15(16):4057. doi: 10.3390/cancers15164057 (PMC10452738; doi:10.3390/cancers15164057)
Supplement: Supplementary file 1 [file cancers-15-04057-s001.zip › supplementary file S4.doc]

**Supplementary file 4.** Sequences of probes, primer sets, and siRNA used in this study

| **Biotin labeled Probe name** | **Sequences (5’ to 3’)** |
| --- | --- |
| hsa_circ_0001380_probe | AACTTCTTCTTGTTTCAAAG |
| hsa_circ_0078607_probe | CAAGGTCAAACTCTGAGTAA |
| hsa_circ_0008305_probe | AACATCTTTTCTGACACAGA |
| β-actin_probe | TTAATGTCACGCACGATTTCCCGCTC |
| **Primer name** | **Sequences (5’ to 3’)** |
| hsa-miR-622 | ACAGTCTGCTGAGGTTGGAGC |
| hsa-miR-1247-3p | CCCCGGGAACGTCGAGACTGGAGC |
| hsa-miR-188-3p | CTCCCACATGCAGGGTTTGCA |
| hsa-miR-1294 | TGTGAGGTTGGCATTGTTGTCT |
| U6 | AGAAGATTAGCATGGCCCCT |
| Universal miRNA Reverse Primer | miRNA First Strand cDNA Synthesis (Tailing Reaction) (Sangon Biotech, Shanghai, China) |
| hsa_circ_0000724 | F: ATCTCTGGGTCCTGCATTTG  R: TATGAGCGCTTCCAGGTCTT |
| hsa_circ_0000918 | F: GGCCCAGCAGAAAAGAGACT  R: AATGAGCCGCAAGTCCAGT |
| hsa_circ_0001072 | F: GACCATTCCCATCAGTGAGC  R: TTGCAGCAAGATGAAAATGC |
| hsa_circ_0001238 | F: CAGGACGAGAAGCTGAAGGA  R: GGCCAGGAATTGAAGAAGGT |
| hsa_circ_0001380 | F: TGCATAAAGGCAGCTTTGAA  R: TCCTGCTTTTGAGGAATTGG |
| hsa_circ_0003247 | F: GCAGAAGCCATTACCAGAGG  R: GCTGCAGCAGACTATCCACA |
| hsa_circ_0003520 | F: GAATGTGGGCAGATGAAGGT  R: GGATAATTTGGCGAAGCAAA |
| hsa_circ_0003570 | F: GCCCTGAAGAAGTGCAAGAG  R: TGAAGAGGGCATTTCCTGTC |
| hsa_circ_0003638 | F: GCAATGAGGACAGCTTGTGA  R: CACTCTGGCCAATCCAAAAT |
| hsa_circ_0004405 | F: GTGAGCACTCTTGGGGAGAG  R: CCAGCATATCCACAGGGAAT |
| hsa_circ_0005309 | F: CCTCTACCTGGTCTGCCGTA  R: AAGAAGTTGAAGGCGGTGTC |
| hsa_circ_0007458 | F: GCAGCAGCAGTTCTACACCA  R: GAAGAGTTTGTTCGCCTTCG |
| hsa_circ_0008494 | F: CCCCTCAATGACCTCCAGTA  R: GTCCCTGTTGCTGCGAGTAT |
| hsa_circ_0069152 | F: GAAGGACCATGCTCAGAAGC  R: CACTGCCTTTTTCTCCCTGA |
| hsa_circ_0078607 | F: AGATCCTGAGACGCATTGCT |
|  | R: ACGATCCTGCCATACCTGTC |
| CPSF3 | F: GCACGTTTACAGCAAGAGGTTGG  R: AAGGTTGGCAGTTTTCCCGTCC |
| GFP | F: TCGTGACCACCCTGACCTAC  R: GTCCTCCTTGAAGTCGATGC |
| DsRed | F: CCCCGACTACATGAAGCTGT |
|  | R: CGGCAGTCTTCTTCTGCATT |
| β-actin | F: CTCCATCCTGGCCTCGCT  R: GCTGTCACCTTCACCGTTCC |
| **sgRNA** | **Sequences (5’ to 3’)** |
| sgRNA-CPSF3 | F: CACCGCCACAAAAGCTATTTATAGA  R: AAACTCTATAAATAGCTTTTGTGGC |
| **miRNA mimics** | **Sequences (3’ to 5’)** |
| hsa-miR-622 | ACAGUCUGCUGAGGUUGGAGC |
| hsa-miR-188-3p | CUCCCACAUGCAGGGUUUGCA |
